# Centriole Duplication at the Crossroads of Cell Cycle Control and Oncogenesis

**DOI:** 10.3390/cells14141094

**Published:** 2025-07-17

**Authors:** Claude Prigent

**Affiliations:** CRBM, Centre de Recherche en Biologie Cellulaire de Montpellier, CNRS—Université de Montpellier, 1919 Route de Mende, 34293 Montpellier, France; claude.prigent@crbm.cnrs.fr; Tel.: +33-(0)631122757

**Keywords:** centriole duplication, cell cycle control, chromosomal instability, oncogenesis, ciliopathies

## Abstract

Centriole duplication is a vital process for cellular organisation and function, underpinning essential activities such as cell division, microtubule organisation and ciliogenesis. This review summarises the latest research on the mechanisms and regulatory pathways that control this process, focusing on important proteins such as polo-like kinase 4 (PLK4), SCL/TAL1 interrupting locus (STIL) and spindle assembly abnormal protein 6 (SAS-6). This study examines the complex steps involved in semi-conservative duplication, from initiation in the G1–S phase to the maturation of centrioles during the cell cycle. Additionally, we will explore the consequences of dysregulated centriole duplication. Dysregulation of this process can lead to centrosome amplification and subsequent chromosomal instability. These factors are implicated in several cancers and developmental disorders. By integrating recent study findings, this review emphasises the importance of centriole duplication in maintaining cellular homeostasis and its potential as a therapeutic target in disease contexts. The presented findings aim to provide a fundamental understanding that may inform future research directions and clinical interventions related to centriole biology.

## 1. Introduction to Centrioles and Centrosomes

Centrioles and centrosomes are essential components of cellular architecture that play a key role in organising the microtubule cytoskeleton, ensuring accurate cell division and facilitating the formation of cilia and flagella [1]. In animal cells, the centrosome is often referred to as the major microtubule organising centre (MTOC) and comprises a pair of centrioles surrounded by an amorphous matrix of proteins known as the pericentriolar material (PCM). The components of the pericentriolar material have a regular disposition; they self-assemble to form a cylindrical scaffold with a mesh-like architecture. It is important to understand that these structures work together to organise the spatial arrangement of microtubules during interphase, as well as directing the formation of the bipolar mitotic spindle during cell division. This is essential for proper chromosome segregation and genomic stability [2]. Centrioles and centrosomes are subject to stringent regulation due to their pivotal role in preserving cellular homeostasis. Defects in their function or duplication have been associated with diseases such as cancer and neurodevelopmental disorders [3,4,5].

### 1.1. Structure of Centrioles

**Figure 1:**
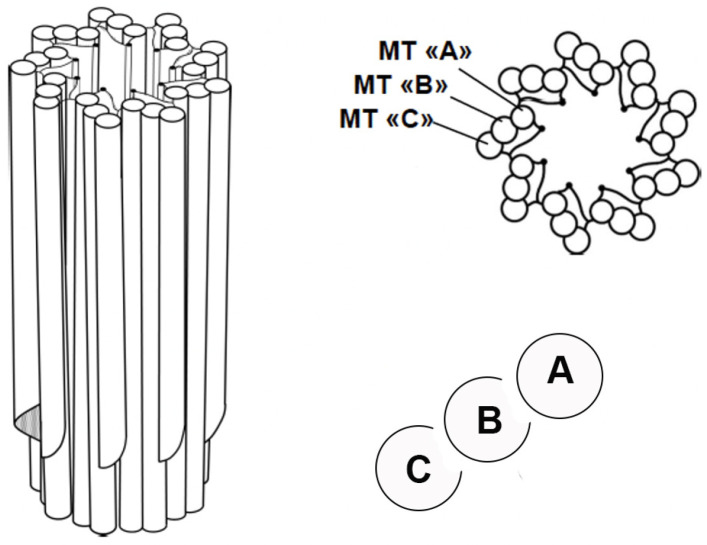
A barrel-shaped centriole displaying nine-fold symmetry and consisting of nine microtubule triplets. ‘A’ represents a complete microtubule, while ‘B’ and ‘C’ represent incomplete microtubules. The triplets are located at the base of the centriole, while the distal end consists of doublets.

Centrioles are barrel-shaped organelles composed of microtubule singlets, doublets or triplets arranged in characteristic nine-fold radial symmetry. Advances in electron microscopy have provided detailed insights into their ultrastructure, revealing their nine-fold symmetry and microtubule triplet arrangement [6]. Each triplet consists of one complete microtubule (the A-tubule) and two incomplete microtubules (the B- and C-tubules) [7] (Figure 1). How these triplets are formed is still the subject of intense research [8]. Centrioles have an approximate diameter of 200 nm and a length of 500 nm. Despite their small size, they serve as platforms for recruiting the proteins required for microtubule nucleation and anchoring, which makes them indispensable for various cellular processes [9].

In most animal cells, centrioles occur in pairs associated by a structure called a linker. The centrioles are oriented orthogonally to each other. They are not identical: one is the ‘mother’ centriole and the other the ‘daughter’ centriole [10].

**Figure 2:**
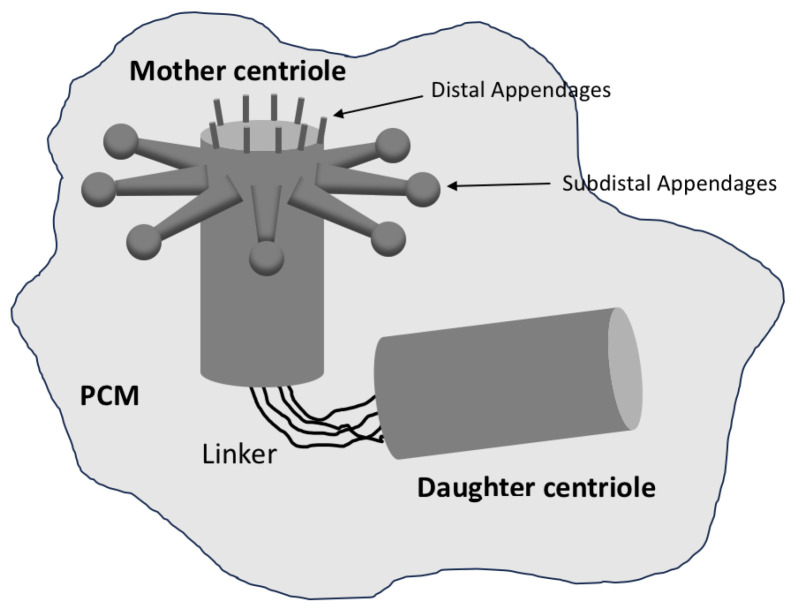
A G1 centrosome consists of a mother centriole with its distal and subdistal appendages, as well as a daughter centriole that lacks appendages. Both are associated with a linker and are surrounded by pericentriolar material (PCM).

As a general rule, the mother centriole is older and more structurally mature than the daughter centriole. The distal end of the mother centriole has appendages involved in anchoring and organising microtubules (see Figure 2). Appendage proteins are also essential for the formation of a cilium or flagellum, assembled on a basal body organised by the mother centriole [11]. In contrast, the daughter centriole lacks these appendages and must mature before acquiring full functionality.

### 1.2. The Centrosome and Its Function

The centrosome is a dynamic organelle that plays a crucial role in the spatial organisation of the microtubule network within cells. In most animal cells, it acts as the main microtubule organising centre and is involved in a variety of processes. It nucleates and anchors microtubules, thereby ensuring that the cell maintains a polarised microtubule array essential for processes such as intracellular transport and cell migration [12].

During cell division, the centrosome duplicates in interphase, with each copy migrating to opposite poles of the cell in prophase of mitosis to form the mitotic spindle. This apparatus is responsible for precisely segregating chromosomes into daughter cells, ensuring that each newly formed cell inherits genetic material identical to that of the parent cell. Errors in centrosome function or duplication can therefore lead to aberrant spindle formation, resulting in chromosomal instability and aneuploidy—hallmarks of many cancers [13,14].

Centrioles are also essential for the formation of cilia and flagella. During this process, the mother centriole adheres to the plasma membrane and transforms into a basal body, which initiates cilia formation [15]. Cilia and flagella are essential for various functions, including cell motility and the movement of fluids across epithelial surfaces. This may be the primary function of centrioles. Research has shown that Drosophila without centrioles can reach adulthood, indicating that cells can divide in their absence. However, these flies die soon after birth due to the absence of cilia [16].

### 1.3. Centriole Duplication: A Brief Overview

Centriole duplication is a highly regulated process that ensures the correct number of centrioles is maintained in each cell. Under normal conditions, a non-dividing cell contains two centrioles, or one centrosome. In proliferating cells, these centrioles must duplicate once and only once per cell cycle, ensuring that the cell contains two centrosomes which can then form a bipolar spindle when it enters mitosis. The bipolarity of the spindle is essential for the segregation of the duplicated chromosomes into two sets. During cell division, each daughter cell receives one set of chromosomes and one centrosome [17].

**Figure 3:**
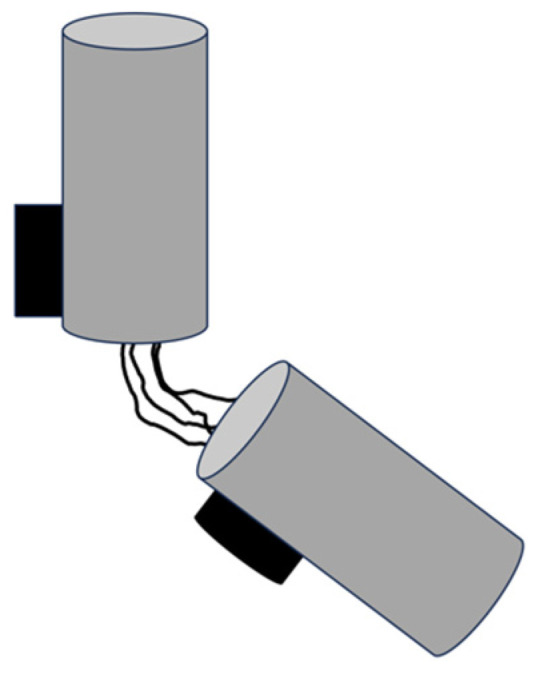
Centriole duplication, which starts with new procentrioles growing at the surface of each old centriole that remains associated by a linker.

The process of centriole duplication is semi-conservative, meaning that a new centriole forms on the surface of an existing one (see Figure 3). Duplication begins late in the G1 phase, just before the transition to the S phase, with the formation of new centrioles that are arranged orthogonally to each parent centriole. This results in cells containing four centrioles (two centrosomes). The duplication process continues during G2 with centriole elongation and maturation [18]. As the cell enters mitosis, the two older centrioles that formed the original centrosome separate, each remaining attached to a new centriole. These two pairs of centrioles then form the centrosomes that organise the poles of the bipolar spindle during mitosis.

### 1.4. The Importance of Centriole Number and Integrity

Proper centriole duplication is essential for maintaining cellular homeostasis and facilitating organism development. Errors in this process can result in centrosome amplification, whereby a single cell contains more than two centrosomes. Centrosome amplification has been shown to disrupt the assembly of the bipolar mitotic spindle, resulting in multipolar spindles and incorrect chromosome segregation. This can contribute to chromosomal instability and is often observed in cancer cells [19]. Conversely, insufficient centriole duplication can hinder the formation of a bipolar spindle, resulting in monopolar spindle assembly, abnormal cell division and aneuploidy [20].

Research has revealed a link between defects in centriole function or duplication and various developmental disorders. For instance, mutations in genes that regulate centriole duplication have been linked to primary microcephaly, a condition characterised by an abnormally small brain due to defects in the division of neural progenitor cells [21]. Furthermore, research has shown that polyploidisation of liver cells, which occurs in the absence of centrioles, can lead to severe liver damage and impaired liver function [22].

### 1.5. Historical Perspective and Discovery of Centrioles

**Figure 4:**
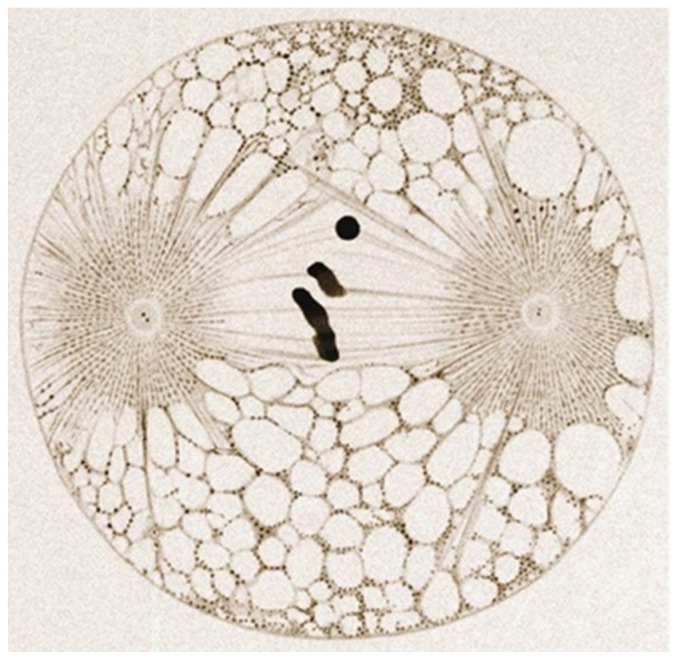
Cell division in *Ascaris megalocephala*, drawn by Theodor Boveri in 1901 from his observations using high optical enlargement.

The discovery of centrioles dates back to the late 19th century, when they were first observed under a microscope by the Swiss anatomist Theodor Boveri [23,24] (Figure 4). Boveri made a significant contribution to our understanding of the role of centrosomes in cell division. For example, he proposed that these structures were essential for ensuring the equal segregation of chromosomes [3].

Until recently, the molecular mechanisms underlying centriole duplication and centrosome function were not well understood. However, advanced molecular biology techniques have now enabled significant progress to be made in this area. Identifying key regulators such as polo-like kinase 4 (PLK4), SCL/TAL1 interrupting locus (STIL) and spindle assembly abnormal protein 6 (SAS-6), and using model organisms such as *C. elegans*, *D. Drosophila*, mice and zebrafish, has improved our understanding of centriole duplication and how its dysregulation contributes to disease [25,26,27].

### 1.6. Relevance of Centriole Research in Disease and Therapeutics

Research into centrioles and centrosomes has significant implications for human health. Centrosome abnormalities are a hallmark of most cancer cells, and the dysregulation of centriole duplication has been associated with tumour formation due to its role in chromosomal instability. Centrosome amplification, driven by the overduplication of centrioles, has been observed in various solid tumours, and its role in cancer progression is the subject of intense investigation [5,28].

Furthermore, defects in centriole duplication have been linked to several genetic disorders, including primary microcephaly and ciliopathies—diseases caused by defective cilia formation [29]. A deeper understanding of the molecular mechanisms that govern centriole duplication could inform effective therapeutic strategies for treating these conditions. For example, PLK4 inhibitors, which are key regulators of centriole duplication, are being investigated as potential cancer treatments to reduce centrosome amplification in tumours [30,31].

Please see below for a summary of the key points.

Centrioles and centrosomes are essential cellular-membrane-less organelles that play a crucial role in the organisation of microtubules, cell division and ciliogenesis. Centriole duplication is a process that is closely linked to DNA replication. Together, these two processes help to maintain genomic stability during cell division. Dysregulation of centriole duplication can have severe consequences, including cancer and developmental disorders. As research in this field advances, a deeper understanding of centriole biology will provide insights into the molecular basis of various diseases and open up new avenues for therapeutic intervention.

## 2. An Overview of the Centriole Duplication Process

Centriole duplication is a highly organised, cell-cycle-dependent process that ensures the correct number of centrioles is maintained within a cell. Typically, a cell contains one pair of centrioles. As the cell cycle progresses, a new procentriole forms on the wall of each existing centriole, initiating centriole duplication. This process is strictly regulated to ensure that it occurs only once per cell cycle, thus guaranteeing that the cell enters mitosis with two centrosomes [32,33]. This section examines the key stages of centriole duplication, the key molecular players involved and the regulatory mechanisms that prevent errors in duplication.

### 2.1. The Centriole Duplication Cycle

**Figure 5:**
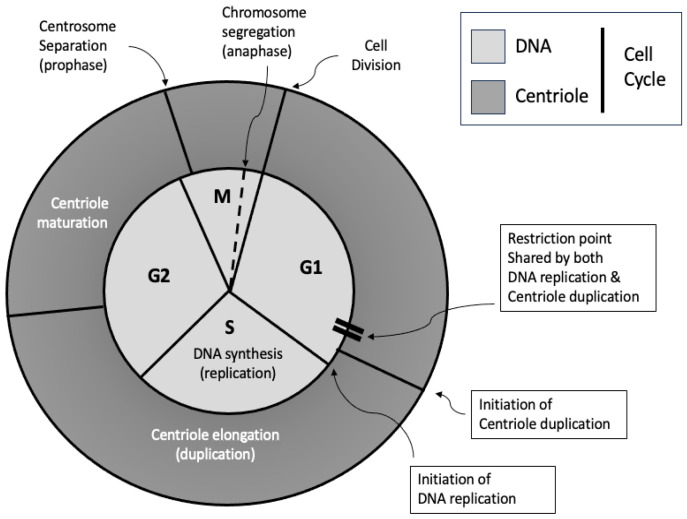
The synchronisation of the DNA cycle (light grey) and the centriole cycle (dark grey). Note that the cell cycle is divided into G1, S, G2 and M phases, which are defined in relation to the DNA cycle.

Centriole duplication occurs in synchrony with DNA replication during the cell cycle. It begins in the late G1 or early S phase and continues through the G2 phase (see Figure 5). Although centriole duplication occurs independently of DNA synthesis, both events are coordinated [34,35,36,37]. This ensures that, before cell division occurs, the cell contains two copies of its genome and precisely two centrosomes. Following cell division, each daughter cell is endowed with one centrosome and one copy of the genome.

Key Phases of Centriole Duplication:

**Initiation** (late G1/early S phase): A new centriole begins to form next to each pre-existing mother and daughter centriole [38].

**Elongation** (S and G2 phases): The new centrioles undergo growth by adding tubulin subunits to their microtubule structures [39,40].

**Splitting:** The linker between the mother and the daughter centrioles is removed before entering mitosis.

**Maturation** (late G2 and M phases): Daughter centrioles mature to become mother centrioles, acquiring full structural and functional capacity [10,41].

**Segregation** (mitosis): During the process of mitosis, the two centrosomes move to opposite poles of the cell [42,43].

### 2.2. Key Stages of Centriole Duplication

#### 2.2.1. Initiation: The Formation of the Procentriole

**Figure 6:**
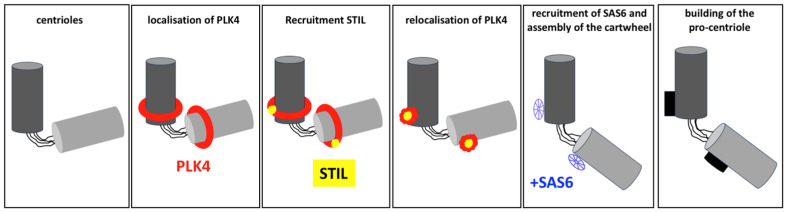
The initiation of centriole duplication, which involves the assembly of the cartwheel through the sequential binding of PLK4, STIL and SAS6.

The first step in centriole duplication is the initiation of procentriole formation, which occurs adjacent to the pre-existing mother centriole [38] (Figure 6). This process begins in the late G1 phase or early S phase of the cell cycle, triggered by the recruitment of several key proteins:

**PLK4**: PLK4 is a master regulator that localises to the surface of the mother centriole, forming a scaffold to recruit other essential proteins, including STIL and SAS-6. It is vital to note that the PLK4 phosphorylation of these proteins is critical for centriole formation [44,45].

**STIL**: STIL interacts with PLK4 and is necessary for recruiting SAS-6, thereby establishing the structural symmetry of the centriole [46].

**SAS-6**: SAS-6 is key to the formation of the cartwheel structure, thereby establishing the nine-fold radial symmetry of the centriole, a process which is essential for its assembly [47].

**Centrosomal Protein 152 and 192** (**CEP152 and CEP192)**: These centrosomal proteins act as scaffolds to recruit PLK4 and other proteins, ensuring that the duplication process is spatially restricted to the site adjacent to the mother centriole [48,49].

Once the recruitment of these proteins has taken place and the cartwheel structure has formed, microtubule assembly begins. This marks the transition to the elongation phase of centriole duplication. The process by which the centriole establishes only one cartwheel during centriole duplication is yet to be explored.

#### 2.2.2. Elongation: The Growth of the Procentriole

Following initiation, the procentriole elongates by incorporating tubulin subunits into the growing microtubule triplets, driven by proteins that regulate tubulin polymerization:

**Centrosomal P4.1-associated protein** (**CPAP**): CPAP promotes the addition of tubulin to the growing microtubules and regulates centriole length by controlling microtubule polymerisation [50].

**Centrosomal protein 135** (**CEP135**): This protein plays a crucial role in stabilising the microtubule triplets that comprise the centriole, thereby ensuring the proper structural integrity during elongation [51].

Elongation continues through the G2 phase of the cell cycle, thereby ensuring that the daughter centriole reaches a length comparable to that of the mother centriole before entering mitosis. Centriole length is controlled by a complex mechanism involving two protein complexes: ‘centriole elongation activators’ (CEAs) and ‘centriole elongation inhibitors’ (CEIs) [40]. Further research is needed to understand how these CEA and CEI complexes work together.

#### 2.2.3. Splitting

**Figure 7:**
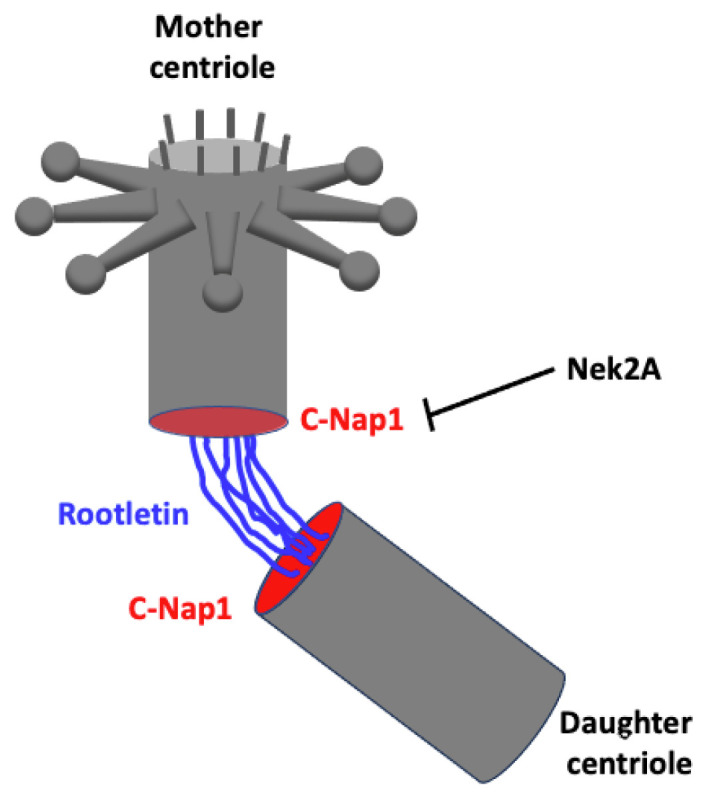
In interphase, mother and daughter centrioles remain associated by a fibrous structure called the linker, which is made up of Rootletin (shown in blue) that is bound to C-Nap1 (shown in red) at the proximal ends of the centrioles. The removal of the linker is achieved through a complex mechanism that mainly depends on C-Nap1 phosphorylation by Nek2A.

Throughout interphase, the mother and daughter centrioles remain connected within the centrosome by a linker that must be removed prior to entry into mitosis to allow separation and ensure bipolar spindle assembly. This linker is a fibrous structure formed by Rootletin proteins that originate from the proximal ends of the centrioles [52]. Rootletin interacts with centrosomal Nek2-associated protein 1 (C-Nap1) (also known as Cep250) at each end of the centrioles [53,54] (Figure 7). Removal of the linker depends on the phosphorylation of C-Nap1 by the NimA-related protein kinase 2 isoform (Nek2A) [55,56]. This is the basic mechanism of centriole cohesion and division, but many other proteins are involved, in particular proteins that interact with both Rootletin and C-Nap1, such as Leucine-rich repeat-containing protein 45 (LRRC45), another substrate of Nek2A [57], or Coiled-coil domain-containing protein 102B (CCDC102B), which interacts with Rootletin and LRRC45 [58].

#### 2.2.4. Maturation: Preparation for Function

**Figure 8:**
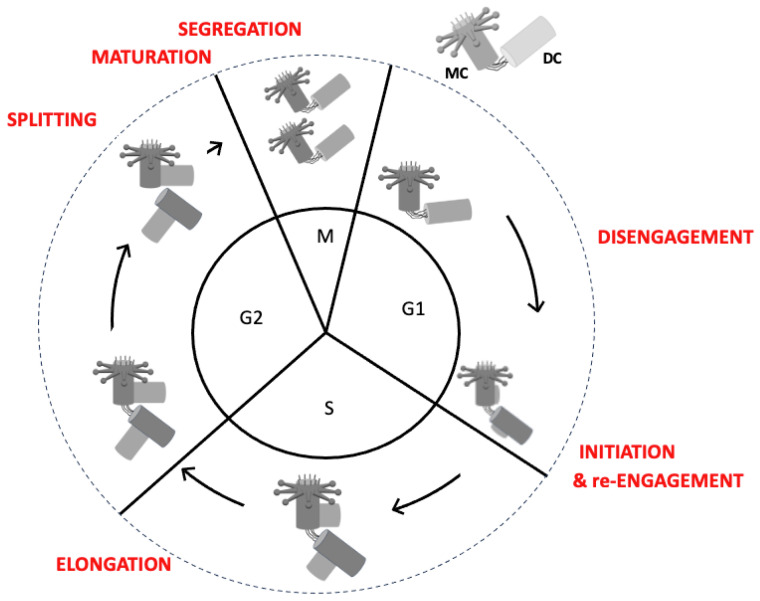
The six stages of centriole duplication during the cell cycle. (DC: daughter centriole; MC: mother centriole). The stages are disengagement, initiation and immediate re-engagement, elongation, splitting, maturation and segregation.

Following elongation, each centrosome contains two centrioles: one with a mother centriole and a daughter centriole, and one with two daughter centrioles (see Figure 8). In the latter case, the maturation of the eldest daughter centriole is essential for it to become a mother centriole. This is crucial for the centrosome to perform all its functions competently. These functions include PCM regulation, which is essential for microtubule nucleation, and acting as a basal body for ciliogenesis [59,60]

What is the most effective method for distinguishing the mother centriole from the daughter centriole? As outlined in reference [61], the mother centriole is structurally distinct and possesses appendages.

What is PCM? The PCM is a dynamic matrix of proteins surrounding the centrosome, the composition of which changes during the cell cycle. For example, during mitosis, when microtubule nucleation is at its peak, the concentration of proteins involved in MT nucleation increases in the PCM [62].

Maturation of the oldest daughter centriole is essential as this prepares the centrosome for subsequent cycles. At the end of mitosis, each daughter cell acquires an active centrosome (comprising a mother centriole and a daughter centriole) that is ready to organise its PCM and initiate a new duplication cycle.

#### 2.2.5. Segregation: Distribution to Daughter Cells

When the cell enters mitosis, the two duplicated centrosomes (each containing a mother centriole and a daughter centriole) move away from each other and travel towards opposite poles of the cell via a microtubule- and actin-dependent mechanism [63]. Nuclear envelope breakdown (NEB) is pivotal in enabling the assembly of the mitotic spindle of microtubules, which are nucleated by the centrosomes and chromosomes. This is essential for chromosome separation into two identical sets. The presence of TWO centrosomes in a mitotic cell is essential for the formation of a BIPOLAR spindle (TWO centrosomes for TWO poles). During the physical division of the two daughter cells, each cell will then inherit an identical set of chromosomes and a centrosome in order to maintain genomic stability [64].

#### 2.2.6. Engagement–Disengagement

Non-proliferating cells have a centrosome composed of two centrioles: a mother centriole and a daughter centriole. These are ‘engaged’, or held together, by cohesion proteins [65]. The small splice variant of Shugoshin 1 (sSgo1) was identified as the first centriole cohesion protein [66]. Engagement–disengagement is a more complex mechanism not described here [67]. It prevents the beginning of centriole duplication and is set up as soon as the daughter centriole appears during duplication. Disengagement occurs upon exit from mitosis through the degradation of sSgo1 by separase, which is activated by a mechanism involving the anaphase-promoting complex (APC/C) [18]. Disengagement coincides with the re-establishment of the linker.

Consequently, the centrioles inherited by the daughter cells are ‘disengaged’, ready for duplication. This disengagement is temporary, however, and is essential to enable a new cycle of centriole duplication during the subsequent cell cycle [18,66].

### 2.3. Mechanisms Preventing Overduplication

A key challenge in centriole duplication is ensuring that each centriole duplicates only once per cell cycle. This is achieved through several regulatory mechanisms [18]. For example, PLK4, which plays a crucial role in the initiation of centriole duplication, is targeted by ubiquitin ligases for proteasomal degradation once initiation has occurred. This process is crucial in preventing overactivation and reduplication [68]. Proteins such as STIL and SAS-6 are also subject to stringent regulation to ensure that aberrant centriole formation is prevented and proper duplication is maintained [69]. As outlined above, when centriole duplication commences, the centrioles become ‘engaged’, meaning ‘locked’, thus preventing the initiation of a new duplication. Once duplication is complete, the centrioles will only be ‘disengaged’ or ‘unlocked’ during the subsequent mitosis. This disengagement ‘licenses’ the mother and daughter centrioles for a new round of duplication in the next cell cycle [18]. As outlined in [70] (Figure 8), these semantics are derived from the DNA replication mechanism.

In conclusion, centriole duplication begins with the initiation of procentriole formation, which is followed by elongation, splitting, maturation and segregation. Key regulatory proteins ensure that duplication occurs precisely once per cell cycle, thereby preventing centrosome amplification and maintaining genomic integrity. Understanding these mechanisms is vital if we are to discover how errors in centriole duplication contribute to diseases such as cancer and neurodevelopmental disorders.

## 3. Key Molecular Players in Centriole Duplication

This section looks in more detail at the key molecules involved in centriole duplication, focusing on PLK4, STIL, SAS-6, CPAP, CEP135 and other helper proteins such as CEP192 and CEP152 that regulate this essential process.

### 3.1. PLK4

PLK4 belongs to the polo-like family of serine/threonine kinases, which comprises five members. All of these proteins are characterised by an N-terminal catalytic domain and at least one polo-box domain at their C-terminal end. As outlined in reference [71], all PLK proteins except PLK5 play a role in controlling cell cycle progression. PLK4 is widely recognised as the master regulator of centriole duplication. It is a serine/threonine kinase that localises to the centrioles, initiating centriole duplication at the end of the G1 phase of the cell cycle. A key function of PLK4 is to phosphorylate target proteins such as STIL and SAS-6, which are essential for forming the nascent centriole [38,69]. In the early stages of centriole duplication, PLK4 phosphorylates STIL, enabling it to interact with SAS-6. This interaction initiates the formation of the centriole cartwheel structure, which is a scaffold required for assembling the core components of the centriole [72,73,74]. As PLK4 is responsible for initiating duplication, its activity levels must be very precisely controlled. Too much PLK4 activity would initiate excessive centriole duplication, which could lead to multipolar spindles and aneuploidy, two hallmarks of cancer cells.

PLK4 is primarily regulated by ubiquitin-mediated proteolysis through several ubiquitin ligases, including βTrCP/Slimb [75], Mib1 [76] and CRL4DCAF1 [77]. The reason why so many ubiquitin ligases target PLK4 remains unclear. As outlined in references [78,79], trans-autophosphorylation of PLK4 is a prerequisite for its subsequent ubiquitination and degradation by the 26S proteasome. Temporal control of PLK4 levels is critical. High levels of PLK4 have been shown to lead to centriole amplification, whereas low levels prevent centriole duplication [33,80]. Autophosphorylation of PLK4 can also promote its own degradation, creating a negative feedback loop that controls centriole number [79]. Therefore, strict regulation of PLK4 ensures that centriole duplication occurs precisely once per cell cycle, thereby avoiding potential abnormalities in centrosome number associated with chromosomal instability and cancer [8,33].

### 3.2. STIL

STIL is a multi-domain protein that contains structured domains, such as coiled-coil and α-helix domains, as well as many intrinsically disordered regions (IDRs). STIL plays a critical role in centriole duplication, a process that depends on its tetramerisation [81]. During the G1 phase of the cell cycle, STIL is recruited to the centrioles, where it is phosphorylated by PLK4 [82]. This phosphorylation enables interaction with SAS-6, facilitating the assembly of the cartwheel structure necessary for the formation of the daughter centriole [83]. STIL acts as a bridge between PLK4 and SAS-6, ensuring centriole duplication occurs in a tightly regulated manner. STIL also plays a vital role in stabilising the cartwheel structure throughout centriole elongation to ensure that the integrity of the developing centriole is maintained during maturation [69].

### 3.3. SAS-6

**Figure 9:**
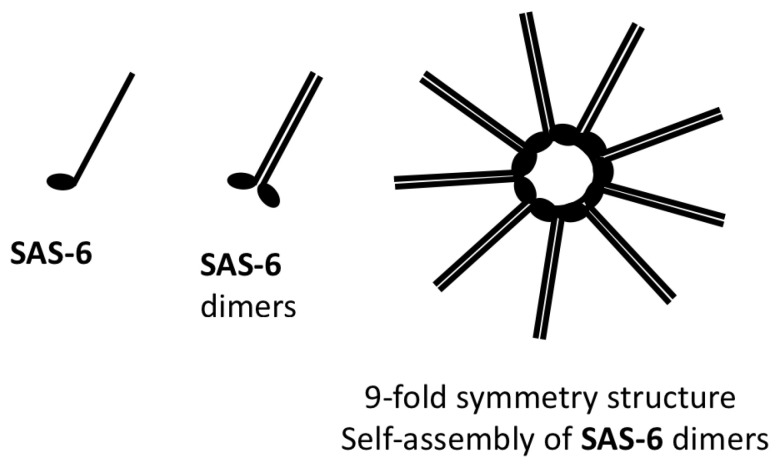
The nine-fold symmetry structure of the cartwheel, detailing the self-assembly of SAS-6 dimers.

SAS-6 is the key component of the cartwheel. It is a coiled-coil protein that can self-assemble into a nine-fold symmetrical structure in vitro [84] (see Figure 9). This structure is located at the centre of the cartwheel and consists of a central hub surrounded by nine SAS-6 spokes [85].

This arrangement is crucial for establishing the nine-spoke symmetry characteristic of centrioles. The cartwheel serves as a scaffold for centriole assembly, providing a model [6,86,87]. The cartwheel thus establishes the fundamental symmetry of the centriole, which is essential for its role as MTOC [87,88]. Importantly, the interaction between SAS-6 and STIL, which is regulated by PLK4 phosphorylation, is essential for centriole duplication [83]. Consequently, SAS-6 plays a crucial role in initiating centriole duplication and maintaining nine-fold centriole symmetry [89].

### 3.4. CPAP

**Figure 10:**
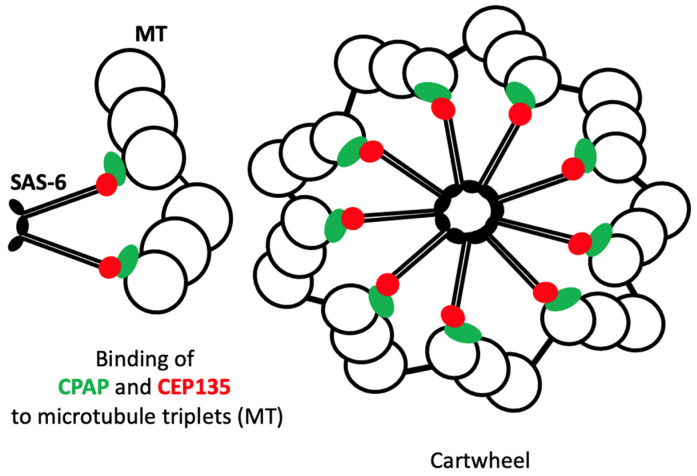
The cartwheel assembly with its nine triplets of microtubules. SAS-6 dimers are shown in black, CPAP in green and CEP35 in red.

CPAP, a centriolar protein that binds to γ-tubulin, is also known as the centromeric protein CENP-J [90,91,92,93]. CPAP regulates centriole elongation by promoting microtubule polymerisation [92]. Following the formation of the cartwheel structure, CPAP is recruited to the growing centriole where it facilitates the addition of tubulin subunits to microtubules. CPAP binds to tubulin dimers, stabilises them and enables their incorporation into the microtubule triplets of the growing centriole [90] (see Figure 10). The regulation of CPAP is crucial, as abnormalities in centriole length can impair spindle formation and potentially contribute to tumour formation. Overexpression of CPAP has been shown to result in centrioles that are excessively elongated, while depletion has been observed to yield centrioles that are significantly shorter and potentially non-functional [50].

### 3.5. CEP135

CEP135 is a scaffold protein that plays a crucial role in centriole formation. It stabilises the microtubule triplets that form the outer structure of the centriole and links the inner cartwheel, formed by SAS-6, to these triplets [93] (see Figure 10). CEP135 plays a vital role in stabilising the centriole during maturation. It interacts with microtubules and other centriolar proteins to ensure the centriole’s structural stability [51]. Mutations in CEP135 have been identified as a factor in defects in centriole structure, which can result in abnormal cell division and developmental disorders [94,95].

### 3.6. Other Proteins Involved in Centriole Biogenesis and Maintenance

It is important to note that CEP192 and CEP152 (centrosomal proteins 192 and 152) are essential for the recruitment of PLK4 during the initial stages of centriole duplication. CEP192 has been shown to act as a scaffold for PLK4, while CEP152 ensures its correct localisation and activation [48,96]. These two proteins are crucial for centriole maturation and, consequently, cell division. A correlation between mutations in these proteins and centrosome dysfunction, with associated diseases including microcephaly has been reported [97]. Auxiliary proteins, such as CEP295 and CEP63, also contribute to centriole duplication by regulating cohesion and the recruitment of structural proteins, as well as controlling centriole length and stability [98,99].

## 4. Regulation of Centriole Duplication

The regulation of centriole duplication involves various mechanisms, including licensing and temporal control, ubiquitination and proteasomal degradation, negative regulation by tumour suppressors like p53 and p21, and mechanisms that prevent reduplication. This section provides a comprehensive exploration of the regulatory pathways in question.

### 4.1. Licensing and Temporal Control

It is imperative that centriole duplication occurs at the correct time during cell cycle progression, and that it is executed with precision and accuracy, occurring only once per cell cycle. This regulation is implemented through a series of checkpoints and regulatory proteins. These proteins ensure that centriole duplication is closely coordinated with the phases of the cell cycle.

#### 4.1.1. Licensing Mechanisms

The G1/S transition is the critical point at which cells are ‘licensed’ to duplicate their centrioles. The timing of this process is crucial, as it ensures that centriole duplication occurs in coordination with DNA replication. This guarantees that the new centrioles are established before subsequent cell division begins. Cyclin-dependent kinases (CDKs), particularly CDK2, play a pivotal role in initiating centriole duplication. During the G1 phase, cyclin E associates with CDK2, facilitating the phosphorylation of key proteins involved in DNA replication [100,101,102]. These phosphorylations are essential for the recruitment of regulatory proteins to the centrosome. Upon entering the S phase, the presence of cyclin E-CDK2 complexes promotes the assembly of new centrioles adjacent to pre-existing mother centrioles [103,104].

#### 4.1.2. Preventing Overduplication

As with DNA, the centrosome must duplicate once and only once per cell cycle. How is this accomplished?

This is achieved by controlling the expression of key proteins, such as STIL and SAS-6, which are essential for centriole assembly. The expression levels of these proteins are therefore tightly regulated to prevent excessive centriole formation. For example, overexpressing STIL results in supernumerary centrioles being present, similar to the effects of overexpressing SAS-6 [105,106,107].

Secondly, feedback loops monitor the integrity of centriole duplication. For instance, the activity of CDKs is regulated by cyclin degradation at the end of mitosis, thus preventing the premature initiation of duplication in the subsequent cell cycle. This process is vital in ensuring that centriole duplication occurs only once and that cellular integrity is maintained [33].

#### 4.1.3. Checkpoints in Centriole Duplication

The G2/M checkpoint plays a key role in ensuring that the cell is ready to enter mitosis. For example, if centriole duplication is incomplete, or if there are issues with DNA replication, the cell cycle will progress more slowly, or even halt, to allow time for necessary repairs [108]. The regulation of centriole duplication is linked to other cell cycle regulatory pathways. One example is the activity of retinoblastoma protein (pRb), a tumour suppressor that affects the G1/S transition. Hypophosphorylated pRb inhibits S-phase entry, whereas hyperphosphorylation by G1 cyclin/CDK complexes triggers it [109,110]. Treating cells lacking pRB (weak G1/S checkpoint) with hydroxyurea has been shown to result in centrosome amplification [111].

### 4.2. Ubiquitination and Proteasomal Degradation

Ubiquitination is a critical post-translational modification that regulates various cellular processes by targeting proteins for degradation. For example, it plays a role in preventing centrosome overduplication. Indeed, PLK4 kinase activity is directly involved in controlling centrosome numbers [44]. Excessive PLK4 activity can result in centriole overduplication, disrupting normal centrosome function and contributing to genomic instability—a hallmark of cancer [33]. PLK4 is widely accepted to be the key regulator of centriole duplication, meaning that its levels must be meticulously controlled. One of the main mechanisms for this control is the degradation of proteins via a pathway dependent on ubiquitination. PLK4 is a suicide kinase. When it undergoes autophosphorylation, it becomes a substrate for the Skp1–Cullin1–F–box protein-*beta*-transducin repeat-containing protein (SCF-βTrCP) ligase complex. This leads to ubiquitination and subsequent degradation by the proteasome [79]. However, it should be noted that SCF-βTrCP is not the only ubiquitin ligase involved in PLK4 degradation. Cullin4A-RING E3 ubiquitin ligase-DDB1-CUL4-associated factor 1 (CRL4^DCAF1^), for instance, has recently been identified as a ubiquitin ligase that targets PLK4 independently of its kinase activity. CRL4^DCAF1^ has been reported to be involved in controlling centrosome numbers [77]. The control of PLK4 degradation creates a self-regulating feedback loop. Centriole duplication is initiated by increased levels of PLK4, which then lead to its own degradation, thus preventing the risk of further rounds of duplication. A comprehensive understanding of PLK4 regulation through ubiquitination is essential for developing effective cancer therapies. Targeting PLK4 for the treatment of cancers has proven to be an efficient strategy [112,113]. Therefore, targeting the pathways that regulate PLK4 could provide new approaches to treating cancers characterised by centrosome amplification. Finally, the E3 ligase TRIM37 was identified in a screen for proteins regulating the number of centrioles in cycling cells. [114] Mutations in TRIM37 lead to MULIBREY nanism, characterised by the development of tumours. TRIM37 targets CEP192 and centrobin but not PLK4 [115]. The identification of TRIM37 targets might help in understanding its function; does TRIM always target proteins for degradation or does it also modify protein through mono ubiquitination?

### 4.3. Negative Regulation by p53 and p21

The tumour suppressor protein p53 plays a key role in the cellular response to stress and DNA damage. It is well established that this process exerts a negative regulatory influence on the cell cycle, thereby ensuring the maintenance of genomic integrity. p53 is activated in response to DNA damage and arrests cell cycle progression. It acts in either the G1 phase to prevent entry into the S phase or in the G2 phase to prevent entry into the M phase in the presence of DNA lesions. One of p53’s functions is to activate the expression of the p21 protein, a cyclin-dependent kinase inhibitor [116]. P21 binds directly to cyclin-CDK complexes. In the case of CDK2, this binding has been found to delay the G1/S transition. This prevents the initiation of DNA replication and centriole duplication. This allows time for damaged DNA to be repaired before the cell enters the S phase.

In the context of stress responses, p53 has been reported to downregulate PKL4 expression, thereby preventing centrosome amplification [117]. However, on the other hand, the loss of p53 can also result in centrosome hyperamplification [118]. p53 prevents genome instability by arresting cell growth in the presence of defects in centriole duplication induced by the absence of PKL4, for example [5]. These regulations are crucial for maintaining proper centriole numbers in response to cellular stress. The regulatory roles of p53 and p21 are critical in tumour suppression [119]. Mutations in p53 are among the most common alterations in cancer, leading to uncontrolled cell proliferation and potential centrosome abnormalities [120]. A comprehensive understanding of the interactions between p53, p21 and centriole duplication could inform the development of effective therapeutic strategies aimed at eliminating cells with amplified centrosomes [121].

### 4.4. Centriole Reduplication Block

Once centriole duplication has been initiated, there are mechanisms in place to prevent reduplication until the next cell cycle begins [122]. During interphase, cohesion proteins link the mother and daughter centrioles together, preventing premature separation and duplication. This connection is essential to ensuring that centrioles do not duplicate until the cell is ready for the next cycle [18,123]. The timing of centriole duplication is also strictly regulated. Once centrioles have been duplicated, specific signals prevent any further duplication until the cell has completed mitosis and returned to interphase [108]. Following centriole duplication, PLK4 activity decreases due to ubiquitination and degradation. This reduction in PLK4 levels effectively silences its activity until the next cell cycle, ensuring that centrioles are not duplicated prematurely [79]. Centriole maturation, which is controlled by PLK1, is also critical in controlling reduplication [124]. During mitosis, the centrosomes, each of which contains a pair of centrioles, act as the poles of the mitotic spindle. The proper separation and function of the centrioles are essential for the accurate organisation of the spindle apparatus and the correct distribution of chromosomes to daughter cells [33]. The coordination of centriole duplication and separation with mitotic events underscores the intricacy of cellular regulation. Disruptions to this coordination can lead to mitotic errors and contribute to aneuploidy, a common feature of cancer [108].

The process of regulating centriole duplication is multifaceted. It involves several mechanisms, such as licensing, ubiquitination pathways and negative regulatory proteins, as well as systems that can be used to block reduplication. Understanding these regulatory pathways is vital if we are to discover how errors in centriole duplication contribute to various diseases, especially cancer. This knowledge could inform the development of targeted therapies aimed at restoring proper cellular function.

## 5. Aberrant Centriole Duplication and Its Consequences

Centriole duplication is a highly regulated process that is vital for maintaining cellular integrity and function. However, dysregulation leads to significant abnormalities, including centrosome amplification. This section examines the processes involved in aberrant centriole duplication, its consequences and its impact on human health, with a particular focus on cancer and developmental disorders.

### 5.1. Centrosome Amplification

Centrosome amplification, defined as the presence of more than two centrosomes, is a common feature of many cancer cells and is associated with various cellular abnormalities.

#### 5.1.1. Dysregulation of Centriole Duplication Proteins

PLK4 is the key regulator of centriole duplication and is essential for initiating the process. Elevated PLK4 levels, whether through overexpression or hyperactivation, has been shown to promote centriole overduplication, resulting in centrosome amplification [125]. This condition is frequently observed in various cancers, where cancer cells exhibit abnormal centrosome numbers. SAS-6 is another essential protein in centriole assembly that contributes to the formation of the cartwheel structure. Elevated levels of SAS-6 have also been shown to promote the assembly of additional centrioles, leading to the development of supernumerary centrosomes [69]. It is well documented that, when coupled with PLK4 dysregulation, the likelihood of centrosome amplification increases significantly. Several other proteins involved in centriole duplication, such as STIL and CPAP, are also critical for maintaining proper centriole numbers. Dysregulation of these proteins has been shown to disrupt the delicate balance of centriole duplication, which can contribute to centrosome amplification [126].

#### 5.1.2. Causes of Amplification

The causes of centrosome amplification are as follows: failure of cell division; a defect in the licensing mechanism that restricts centriole duplication; or centrosome overduplication [127,128,129]. Mutations in genes that regulate centriole duplication are a significant cause. These mutations can alter the function of proteins, resulting in a loss or gain of function that affects centriole duplication. For example, a gain-of-function mutation in the PLK4 gene leads to the unregulated initiation of centriole formation and centrosome amplification [33]. A large analysis of The Cancer Genome Atlas (TCGA) genomic and transcriptomic data focusing on the ‘centrosome-ome’ identified MCPH1/microcephalin loss as a major cause of centriole amplification, involving an increase in STIL [130]. It is well established that the oncogene c-Myc can drive the expression of centriole duplication proteins [131]. Other oncogenes, that affect cell cycle progression, can cause centrosome amplification. For instance, overexpression of AURKA, seen in cancer cells, causes centrosome amplification due to a defect in cell division [127,132]. The integrity of the cell cycle checkpoints is crucial for proper centriole duplication. Failure of these checkpoints, particularly during the G1/S and G2/M transitions, can lead to excessive centriole duplication. For example, if the G1/S checkpoint fails, cells may enter the S phase with an excessive number of centrosomes, resulting in an amplification phenotype [122].

Centrosome amplification is driven by the dysregulation of centriole duplication proteins and is exacerbated by genetic mutations, oncogene activation and checkpoint failure. This has considerable implications for cellular function and organismal health because centrosome amplification contributes to the oncogenic processes [27].

### 5.2. Cellular Consequences

The consequences of centrosome amplification are significant and wide-ranging, particularly with regard to cell division and genomic stability.

#### 5.2.1. Formation of Multipolar Spindles

The presence of additional centrosomes results in the formation of multipolar spindles during mitosis. In normal mitosis, two centrosomes organise a bipolar spindle to ensure proper chromosome segregation. However, cells with more than two centrosomes can develop multipolar spindles, resulting in improper chromosome alignment and segregation [133]. It is well documented that multipolar spindles significantly increase the risk of chromosome mis-segregation. The additional spindle poles can pull chromosomes in multiple directions, resulting in the unequal distribution of genetic material during cell division. This mis-segregation can result in aneuploidy, a condition in which cells possess an abnormal number of chromosomes [33].

#### 5.2.2. Cancer and Chromosomal Instability

Centrosome amplification can lead to severe cellular consequences, including the formation of multipolar spindles and chromosome mis-segregation. This aneuploidy, which results from centrosome amplification, is a hallmark of cancer cells and contributes to chromosomal instability. Furthermore, cells with abnormal chromosome numbers often exhibit increased mutation rates, driving tumour progression and metastasis [134]. This genomic instability can therefore help cells acquire additional mutations that give them a survival advantage in the tumour microenvironment. Centrosome amplification clearly contributes to tumour development by promoting cell proliferation and survival [25,27]. This amplification can therefore provide a selective advantage, enabling cancer cells to proliferate uncontrollably [135,136].

### 5.3. Microcephaly and Developmental Defects

Aberrant centriole duplication is a feature not only of cancer, but also of developmental disorders, particularly primary microcephaly [137]. This condition is characterised by reduced brain size and cognitive impairments.

Mutations in genes involved in centriole duplication, such as STIL and CPAP, are directly linked to primary microcephaly. These mutations disrupt normal centriole formation and function, resulting in defects in neuronal progenitor cell division and brain development [138].

Aberrant centriole duplication has also been linked to a range of developmental defects, including ciliopathies, which are syndromes involving impaired cilia function. Defective cilia can result in various health issues, including developmental delays and organ malformations. This emphasises the critical role of centrioles in normal development [4,139]. Aberrant centriole duplication can have far-reaching consequences, including centrosome amplification, chromosomal instability and developmental disorders. Evidently, the dysregulation of proteins involved in centriole duplication, genetic mutations and checkpoint failures contributes to these abnormalities. Understanding these mechanisms and assessing their impact on human health is vital. This is particularly important in order to develop effective strategies to combat cancer and developmental disorders.

## 6. Centriole Duplication and Disease

Centriole duplication is thus a fundamental cellular process, and its dysregulation is associated with several diseases [140]. Indeed, a deeper understanding of the relationship between centriole duplication and these diseases could offer valuable insights into their underlying mechanisms and identify potential therapeutic targets.

### 6.1. Cancer

Centrosome amplification is a common feature of cancer cells and is often correlated with increased tumour aggressiveness [141]. Between 30 and 40% of solid tumours exhibit this phenomenon, which can lead to genomic instability and cancer progression [33]. So, is centrosome amplification a cause or a consequence of cancer? Evidence suggests that it is both. Not only do cancer cells show centrosome amplification, they can also contain extra-long centrioles [142]. As said before, there is a clear link between centrosome amplification and tumorigenesis [27].

The MYC oncogene, which is known for its role in regulating cell proliferation and growth, is responsible to centrosome amplification when overexpressed [143]. As previously mentioned, this effect is due to transcription activation of genes involved in centriole duplication.

The tumour suppressor p53 plays a critical role in maintaining genomic integrity by regulating the cell cycle. Under normal conditions, p53 induces cell cycle arrest in response to DNA damage, thereby preventing DNA replication and centriole duplication. Clearly, cells lacking p53 divide with an excess of centrosomes [127,144]. As is well documented, loss of p53 function, a common occurrence in many cancers, results in unchecked centriole duplication and subsequent centrosome amplification.

It is important to note that other oncogenes and tumour suppressors can also influence centrosome dynamics. For instance, the loss of function of tumour suppressors such as the adenomatous polyposis coli (APC) is an example of this phenomenon [145].

Mutations in the Ras pathway can also lead to enhanced centriole duplication in skin cancer. However, it is notable that this only occurs in the presence of p53 gain-of-function mutants and not in its absence [146].

#### Therapeutic Targeting

Targeting the machinery involved in centriole duplication is a novel approach to cancer treatment. As centrosome amplification has been observed in almost all types of cancer and centrosomes cannot duplicate in the absence of PLK4, PLK4 has emerged as a key target [147]. And indeed, PLK4 inhibitors could selectively reduce centrosome amplification and subsequent aneuploidy, making them attractive candidates for targeted cancer therapies [33]. Following the report of its depletion by RNA-silencing-induced apoptosis, PLK4 emerged as a promising target for cancer treatment [148]. Preclinical studies have produced highly encouraging results, indicating that PLK4 inhibition could be a valuable addition to cancer treatment [149,150]. In a professional context, combining PLK4 inhibitors with chemotherapeutic agents such as sorafenib has been shown to result in a synergistic anti-tumour effect in vitro. This finding suggests that the combination may enhance treatment efficacy [151]. It was reported that using PLK4 inhibitors could improve the efficacy of cancer treatments by reducing the number of centrosomes, thereby enhancing patient outcomes [31]. Synthetic lethal relationships can also help to select cancers for treatment with PLK4 inhibitors. For example, breast cancers and neuroblastomas that overexpress TRIM37 are highly sensitive to PLK4 inhibition [152].

Centriole duplication dysregulation has been shown to significantly impact cancer progression, with centrosome amplification being a critical factor. Oncogenes and tumour suppressors such as MYC and p53 play vital roles in regulating this process. Targeting the centriole duplication machinery therapeutically, particularly through the use of PLK4 inhibitors, shows great potential for developing future cancer therapies.

### 6.2. Neurological Disorders

Microcephaly, a condition characterised by an abnormally small head size and associated developmental delays, has been linked to mutations in centriole-related proteins [153]. Studies have demonstrated that mutations in genes such as STIL, CPAP and KIFC1 can disrupt the normal process of centriole duplication, resulting in impaired neurogenesis [138]. These mutations have been shown to result in a decrease in neural progenitor cells, which can subsequently affect brain size and development [154,155]. Defects in centriole duplication can cause problems during cell division, resulting in improper mitotic spindle function and the subsequent loss of neuronal progenitors. Studies have shown that a reduction in centriole number directly correlates with decreased cell division in the developing brain, resulting in microcephaly [156]. Fully functional centrioles (also known as ‘mother centrioles’) play a crucial role in ciliogenesis, particularly in the formation of primary cilia. This is important for neurogenesis [157]. A link has been reported between autism spectrum disorders (ASDs) and ciliopathies, particularly orofaciodigital syndrome I (OFD1) [158]. The OFD1 gene codes for a protein that plays a crucial role in regulating centriole length and cilium formation. Genetic analysis has revealed mutations in centriole-associated genes in individuals diagnosed with ASD [159].

Defects in centriole duplication have been identified as a contributing factor in several neurological disorders, including microcephaly and other neurodevelopmental conditions. Furthermore, it was suggested that mutations in centriole-related proteins can disrupt normal brain development, resulting in significant cognitive and developmental challenges.

### 6.3. Ciliopathies

Ciliopathies are a diverse group of genetic disorders caused by defects in the formation and function of cilia, which are often linked to impaired centriole duplication. Centrioles act as the basal bodies that anchor cilia and are essential for their proper assembly and function. Dysregulation of centriole duplication has been shown to result in abnormal or dysfunctional cilia formation. These can disrupt signalling pathways that are critical for various cellular processes [160]. Cilia are vital for sensing environmental signals and regulating cellular responses. Impaired ciliogenesis due to defects in centriole duplication can affect pathways such as Hedgehog and Wnt signalling, leading to a wide range of developmental and physiological abnormalities [161,162]. Ciliopathies are associated with disorders such as polycystic kidney disease (PKD), characterised by the formation of cysts in the kidneys. Abnormalities in centriole function and subsequent cilia defects contribute to the pathogenesis of PKD [163]. Another example is Bardet–Biedl syndrome (BBS), which involves obesity, retinal dystrophy and polydactyly. A link has been reported between mutations in centriole-related proteins that impair ciliogenesis and this disorder, thus demonstrating the importance of centriole function in maintaining ciliary integrity [164].

Ciliopathies demonstrate the vital role of centrioles in cilia formation and function. Defects in centriole duplication can result in impaired cilia, leading to significant developmental disorders and diseases, including PKD and BBS.

## 7. Recent Advances in Visualising Centriole

In recent years, technological innovations and novel discoveries have driven significant advancements in our understanding of centriole duplication. This section highlights key developments in high-resolution structural analysis, the identification of new regulatory pathways and the integration of centriole duplication with other cellular processes.

Cryo-electron microscopy (cryo-EM) has transformed structural biology, enabling researchers to visualise biological macromolecules with unparalleled clarity. Although cryo-EM was used as early as 1997 to reconstruct the centrosome cycle [165], this technique has seen a tremendous evolution, with new flash-freezing methods and new detectors producing high-resolution images. cryo-EM has been pivotal in elucidating the intricate architecture of centrioles. For example, the cartwheel structure of centrioles and the arrangement of proteins such as SAS-6 and the microtubule triplets that comprise the centriole have successfully been visualised [166]. Advances in cryo-EM have also facilitated the visualisation of proteins involved in centriole stability. For example, this is the case for the microtubule-associated protein SSNA-1 (Sjögren’s syndrome nuclear autoantigen 1), which can self-assemble to form antiparallel coiled-coils that stabilise microtubule-based structures, such as centrioles. [167].

Understanding these structural interactions at the molecular level is vital if we are to decipher how centriole duplication is initiated and regulated.

Electron cryo-tomography (cryo-ET) has also been instrumental in visualising the components that maintain the microtubule (MT) triplets that make up the centriole [168]. More recently a combination of cryo-EM, cryo-ET and Alfafold2 prediction software was used in a beautiful work to decipher the structure of the γ-TuRC in the centrosome and how it associates with the PCM and the centriole [169].

Microscopy techniques such as STED (stimulated emission depletion) and SIM (structured illumination microscopy) have enabled centrioles to be visualised at resolutions beyond the diffraction limit of light. This has also revealed the nine-fold structure of centrioles and how proteins localise around them during the cell cycle [170,171].

Expansion microscopy (exM) involves expanding the sample and subsequently observing it under a microscope using fluorescent lighting. Laporte and his colleagues produced a visually appealing piece of work illustrating the architecture of a human centriole. This work demonstrates the power of exM [172].

Once again, a combination of exM and STED, as well as cryoEM and cryoET, allowed the authors to visualise and study the organisation of centromeres in *C. elegans*, representing a significant breakthrough! [173].

These techniques have provided valuable insights into the dynamic behaviour of centrioles and centriolar proteins during the cell cycle. Specifically, they have revealed a correlation between structural changes and the location of these proteins.

## 8. Future Directions in Centriole Duplication Research

As research into centriole duplication continues to evolve, several critical areas warrant further investigation. One of the most important questions in this field of research is how centriole duplication is precisely timed and coordinated with the various events that occur during the cell cycle. Although significant progress has been made in identifying the phases during which centriole duplication occurs, the underlying mechanisms that ensure this timing are still not fully understood. How do cells integrate signals from various cell cycle events to influence centriole duplication? Another area of interest is the role of external signals, such as growth factors or stress conditions, in modulating the timing of centriole duplication. A deeper understanding of how environmental cues influence the centriole duplication cycle could have significant implications for developmental biology and cancer research. This enhanced knowledge could reveal potential intervention points.

One of the main challenges in this field is identifying the fundamental principles conserved in all cells that control centriole duplication. Biologists use many different model organisms, with hundreds of normal and pathological cell types. As with the identification of cell cycle controls, this cellular diversity adds complexity to the task, but using this complexity to identify conserved events could be the way forward. How many proteins are needed to reconstitute a centriole in vitro?

Despite significant advancements in identifying key players in centriole duplication, many unknown proteins and pathways are likely to remain to be discovered. The involvement of a large number of ubiquitin ligases in centriole duplication could open up a whole new field of research. When do they intervene in the cycle, and do they commit to mono- or polyubiquitination? What are their targets? Are these targets ubiquitinated, Neddylated, SUMOylated, etc.? Do they act during stress or under normal conditions?

New and increasingly powerful proteomic analysis techniques continue to expand our knowledge of the proteins present in the centrosome [174]. Recent studies combining proteomics and transcriptomics have also revealed splicing proteins associated with the centrosome [115,175]. These results can be benchmarked against those recently obtained from an analysis of the interactome of the centrosomal kinase, Aurora-A, which identified a new function in RNA splicing [176]. Identifying splicing events required for centriole duplication is not an easy task but should be useful.

Future research in centriole duplication is expected to provide valuable insights that could improve our understanding of cell biology and its impact on health and disease. To make progress in this field, we must address unanswered questions about timing and regulation, overcome technological imaging challenges and explore therapeutic implications.

As research into the complexities of centriole duplication continues, the potential for novel therapeutic strategies will become clearer, paving the way for innovative treatments for cancer, ciliopathies and neurological disorders.

## Data Availability

No new data were created or analysed in this study.

## Abbreviations

APC	adenomatous polyposis Coli
APC/C	anaphase-promoting complex
ASD	autism spectrum disorders
AURKA	Aurora A kinase
BBS	Bardet–Biedl syndrome
βTrCP	*beta*-transducin repeat-containing protein
CCDC102B	Coiled-coil domain-containing protein 102B
CDK(s)	cyclin-dependent kinase(s)
CDK2	cyclin-dependent kinase 2
CEAs	centriole elongation activators
CEIs	centriole elongation inhibitors
CENP-J	centromeric protein J
CEP63	Centrosomal Protein 63
CEP135	Centrosomal Protein 135
CEP152	Centrosomal Protein 152
CEP192	Centrosomal Protein 192
CEP250	Centrosomal Protein 250
CEP295	Centrosomal Protein 295
C-Nap1	Centrosomal Nek2-associated protein 1
CPAP	Centrosomal P4.1-associated protein
CRL4	cullin4A-RING E3 ubiquitin ligase
Cryo-EM	cryo-electron microscopy
DCAF1	DDB1-CUL4-associated factor 1
DC	daughter centriole
DNA	deoxyribonucleic acid
exM	expansion microscopy
γ-TuRC	γ-Tubulin Ring Complex
IDRs	intrinsically disordered regions
KIFC1	kinesin family C1
LRRC45	Leucine-rich repeat-containing protein 45
MC	mother centriole
Mib1	Mindbomb Homologue-1
MTOC	microtubule organising centre
Myc	myelocytomatosis oncogene
Nek1A	NimA-related protein kinase 2
NimA	never in mitosis A
OFD1	orofaciodigital syndrome I
p53	protein 53 (or TP53 for tumour protein 53)
p21	protein 21 (or CIP1 for cyclin-dependent kinase inhibitor 1)
PCM	pericentriolar material
PKD	polycystic kidney disease
PLK1	polo-like kinase 1
PLK4	polo-like kinase 4
PLK5	polo-like kinase 5
pRb	retinoblastoma protein
RNA	ribonucleic acid
SAS-6	spindle assembly abnormal protein 6
SCF	Skp1–Cullin1–F–box protein
sSgo1	Shugoshin 1
SIM	structured illumination microscopy
SSNA-1	Sjoegren syndrome nuclear autoantigen 1
STED	stimulated emission depletion
STIL	SCL/TAL1 interrupting locus
TRIM37	Tripartite motif-containing protein 37
